# Bipolar disorder with comorbid anxiety disorders: impact of comorbidity on treatment outcome in cognitive-behavioral therapy and psychoeducation

**DOI:** 10.1186/2194-7511-1-15

**Published:** 2013-09-02

**Authors:** Lisa D Hawke, Vytas Velyvis, Sagar V Parikh

**Affiliations:** Université de Saint-Boniface, Suite 2237, 200 Cathedral Avenue, Winnipeg, Manitoba R2H 0H7 Canada; York University, Toronto, Canada; Adler International Learning, Toronto, Canada; University Health Network, Toronto, Canada; University of Toronto, Toronto, Canada

**Keywords:** Bipolar disorder, Anxiety disorders, Comorbidity, Cognitive-behavioral therapy, Psychoeducation

## Abstract

**Background:**

Comorbid anxiety disorders are extremely prevalent in bipolar disorder (BD) and have substantial impact on the course of illness. Limited evidence regarding treatment factors has led to a renewal of research efforts examining both the impact of treatments on comorbid anxiety and the impact of comorbid anxiety on treatments. The current study examines the impact of comorbid anxiety disorders on response to two psychosocial interventions for BD.

**Methods:**

A sample of 204 patients with BD took part in the study. Of them, 41.7% had a comorbid anxiety disorder. All participants received either individual cognitive-behavioral therapy or group psychoeducation for BD. Evaluations included complete pretreatment and 18-month follow-up assessments of mood and anxiety symptoms, functioning, medication compliance, dysfunctional attitudes, and coping style. Outcome was compared based on the presence or absence of a comorbid anxiety disorder.

**Results and discussion:**

The participants with comorbid anxiety disorders ranked more severe than those without on several measures. Despite more severe illness characteristics, the magnitude of their treatment gains was equivalent or superior to that of the participants without anxiety disorders on a variety of outcome measures. Although the treatments did not specifically target the anxiety disorder, the participants made significant improvements in anxiety symptoms. Despite greater illness severity, patients with comorbid anxiety disorders can make substantial gains from psychosocial interventions targeting BD. Even in the presence of an anxiety disorder, they are able to attend to the content of the psychosocial treatments and apply it to better manage their condition. The presence of a comorbid anxiety disorder should not be considered a deterrent to offering BD-focused psychosocial treatments.

## Background

Bipolar disorder (BD) is a complex, chronic mood disorder involving repeated episodes of depression and mania/hypomania (American Psychiatric Association 2001). In addition to multiple relapses of mood episodes (Schaffer et al. 2006), individuals living with BD also experience substantial symptoms between episodes (Benazzi 2004; Paykel et al. 2006).

Complicating the course of illness, psychiatric comorbidity is very highly prevalent (Schaffer et al. 2006). Anxiety disorders are among the most highly prevalent comorbidities associated with BD. Epidemiological studies show that as many as 74.9% of individuals with BD have at least one anxiety disorder at some point in their lives (Merikangas et al. 2007). Unfortunately, comorbid anxiety disorders are associated with more affective relapses, increased suicidality, sleep disturbances, decreased work and social functioning, and increased barriers to effective treatment (Hawke et al. 2013; Freeman et al. 2002). Furthermore, they appear to reduce the effectiveness of mood stabilizers (Keller 2006) and increase nonadherence to pharmacotherapy (Perlis et al. 2010), substantially complicating the pharmacological treatment approach (Schaffer et al. 2012).

A recent review of publication trends revealed that although the characteristics of the bipolar-anxiety comorbidity set have been described in detail in the literature, there is a paucity of research examining its treatment factors (Provencher et al. 2012). Several studies have suggested that comorbid anxiety disorders interfere with treatment response (El-Mallakh and Hollifield 2008). However, treatment research has largely consisted of small case studies and preliminary reports, with few large-scale trials. Furthermore, most research has addressed pharmacotherapy, surprisingly few examining psychosocial interventions (Provencher et al. 2012; Provencher et al. [Bibr CR28][Bibr CR29]).

Nevertheless, the potential benefits of psychosocial interventions for comorbid anxiety have been discussed, and three main approaches described. The first is an integrated treatment approach, where anxiety-focused techniques are built into a BD-focused treatment, producing moderate results (Williams et al. 2008; Dusser et al. 2009). A second approach is sequential, i.e., targeting the BD and anxiety disorder with two specific, separate interventions; pilot tests of this approach have also produced moderate results (e.g., Mueser et al. 2004). A third approach is a compromise between the two, i.e., the modular treatment of comorbidities within a standard CBT protocol (Otto and Reilly-Harrington 2002; Thienot et al. 2013). However, no studies have been identified that test this approach.

The limited evidence regarding the impact of comorbid anxiety disorders on psychosocial treatments and, conversely, the impact of psychosocial treatments on comorbid anxiety disorders led Provencher et al. (2012; 2011a, b) to call for a renewal of research efforts examining the reciprocal relationship between comorbid anxiety and BD treatments.

Further to those recommendations, this study is a secondary analysis of data from a randomized controlled trial comparing cognitive-behavioral therapy (CBT) with psychoeducation for BD (Parikh et al. 2012). The primary findings of that study were that the two interventions have equivalent impacts on affective symptoms and relapses. This study reanalyzes the data to determine the impact of comorbid anxiety disorders on treatment response.

## Methods

### Participants

A total of 204 participants took part in the study. The average age was 40.9 years (SD = 10.7). The participants had type I BD (BD-I, 72.1%) or type II BD (BD-II, 27.9%), confirmed with the *Structured Clinical Interview for DSM-IV Axis I Disorders* (SCID-I) (First et al. 2002). The inclusion criteria included having at least two episodes of significant symptoms or full episodes within the previous 3 years but no episode in the month preceding randomization. The exclusion criteria included current substance dependence, severe borderline personality disorder, antisocial personality disorder, life-threatening medical illness, acute suicidality or homicidality, or significant cognitive deficits or language problems.

### Procedure

A detailed description of the study is provided elsewhere (Parikh et al. 2012). The participants were randomized, without regard to illness severity, to individual CBT or group psychoeducation for BD and treated on an outpatient basis. Psychoeducation consisted of six didactic 90-min sessions drawn from a published manual (Bauer and McBride 2003). The topics included illness recognition, treatment approaches, coping strategies, and the creation of personal action plans for depression and mania. CBT consisted of 20 individual 50-min sessions from a published manual (Lam et al. 1999). The content included psychoeducation on BD, a relapse action plan, and additional cognitive-behavioral strategies such as behavioral self-monitoring and cognitive restructuring. Evaluations were conducted by trained, experienced research assistants. The number of sessions attended did not differ based on the presence or absence of a comorbid anxiety disorder (*t*(202) = -0.761, *p* = 0.45). The follow-up period was 18 months, and 160 participants provided follow-up data. The study was approved by the research ethics board of each participating medical center, i.e., the University Health Network Research Ethics Board, the Centre for Addiction and Mental Health Research Ethics Board, the Douglas Mental Health University Institute Research Ethics Board, the St. Joseph's Health Care Research Ethics Board, and the UBC Hospital Research Ethics Board. Written informed consent was obtained from all participants.

### Measures

The diagnoses were established using the SCID-I (First et al. 2002). The symptoms were assessed using the *Longitudinal Interval Follow-up Evaluation* scale scores for mania/hypomania, depression, and panic disorder (LIFE) (Keller et al. 1987), the *Clinician-Administered Rating Scale for Mania* (CARSM) (Altman et al. 1994), and the 21-item *Hamilton Rating Scale for Depression* (HAMD) (Hamilton 1967). A specific anxiety scale was not employed in the study; however, since the HAMD contains four items measuring anxiety (psychic anxiety, somatic anxiety, hypochondriasis, and obsessive-compulsive symptoms), these items were extracted on a *post hoc* basis to create a HAMD-Anxiety scale. The 17-item HAMD total was calculated with and without the anxiety items, for a classic HAMD-17 score and a reduced HAMD-Depression measure that explicitly excludes the anxiety component. Additional measures included the *Global Assessment of Functioning* (GAF) (American Psychiatric Association 2001), the *Social Adjustment Scale* (SAS) (Bauer et al. 2001; Glazer et al. 1980), the *Dysfunctional Attitudes Scale* (DAS) (Weissman and Beck 1980), the *Coping Inventory for the Prodromes of Mania* (CIPM) (Wong and Lam 1999), and a single-item rating scale measuring medication compliance.

### Analyses

Pretreatment and posttreatment analyses were conducted to compare the participants with and without comorbid anxiety disorders. For weekly LIFE symptom scores, a mean score was calculated for the first 4 weeks and the last 4 weeks of the study to provide robust symptom measurements. The mixed linear model was employed, with an unstructured covariance matrix, to manage dropouts and missing data. The analyses were conducted using SPSS-21, with a significance criterion of 0.05.

## Results

The comorbid anxiety disorders were identified in 85 participants (41.7%). These included social phobia (36.5% of the anxiety disorder subsample), panic disorder with or without agoraphobia (31.8%), agoraphobia without panic disorder (4.7%), specific phobia (11.8%), obsessive-compulsive disorder (11.8%), posttraumatic stress disorder (10.6%), generalized anxiety disorder (10.6%), and anxiety disorder not otherwise specified (2.4%)^a^. There was no significant difference in age (*t*(202) = 0.018, *p* = 0.99), sex (*χ*^2^(1) = 2.814, *p* = 0.09), marital status (*χ*^2^(2) = 2.975, *p* = 0.23), and primary diagnosis (BD-I vs. BD-II; *χ*^2^(1) = 1.058, *p* = 0.30) based on the presence of a comorbid anxiety disorder.

The treatment impact on mood and anxiety symptoms based on the presence of a comorbid anxiety disorder is presented in Table 1. Significant time effects show that over 18 months, the participants improved significantly in depressive, manic/hypomanic, and anxiety symptoms, with a nonsignificant trend toward improvement on the LIFE-Panic scale. Significant anxiety disorder effects show that the participants with comorbid anxiety disorders ranked significantly more severely on HAMD-Depression and both measures of anxiety symptoms.

**Table 1:** **Treatment impact on mood and anxiety symptoms based on anxiety disorder comorbidity**

	No anxiety disorder		Comorbid anxiety disorder		Time effect		Anxiety disorder effect		Time × anxiety disorder interaction	
	Baseline	18 months	Baseline	18 months						
	***M*** (SD)	***M*** (SD)	***M*** (SD)	***M*** (SD)	***F***	***p***	***F***	***p***	***F***	***p***
Depression										
LIFE-Depression	2.28 (1.43)	1.94 (1.23)	2.84 (1.55)	1.94 (1.35)	*20.16*	*<0.001*	2.66	0.11	*4.36*	*0.04*
HAMD-17	5.82 (4.38)	4.18 (4.88)	8.39 (5.27)	5.02 (6.05)	*19.96*	*<0.001*	*7.49*	*0.01*	2.35	0.13
HAMD-Depression	4.97 (3.69)	3.30 (3.80)	6.54 (4.29)	3.83 (5.04)	*22.54*	*<0.001*	*4.34*	*0.04*	1.21	0.27
Mania										
LIFE-Mania	1.39 (0.87)	1.17 (0.49)	1.41 (1.01)	1.15 (0.46)	*8.31*	*0.004*	0.01	0.94	0.06	0.81
CARSM	1.80 (2.61)	1.32 (3.16)	2.35 (3.71)	1.19 (3.19)	*5.18*	*0.024*	0.32	0.58	0.91	0.34
Anxiety										
LIFE-Panic	1.01 (0.08)	1.02 (0.12)	1.25 (0.55)	1.09 (0.40)	3.35	0.07	*16.40*	*<0.001*	*4.08*	*0.047*
HAMD-Anxiety	1.02 (1.40)	0.93 (1.45)	2.14 (1.80)	1.26 (1.70)	*8.01*	*0.005*	*14.75*	*<0.001*	*5.41*	*0.021*

Significant effects are italicized. *CARSM* Clinician-Administered Rating Scale for Mania, *HAMD*-*17* Hamilton Rating Scale for Depression, 17-item version, HAMD-Anxiety, Hamilton Rating Scale for Depression, four anxiety items only, *HAMD*-*Depression* Hamilton Rating Scale for Depression, excluding anxiety items, *LIFE* Longitudinal Interval Follow-up Examination.

Significant anxiety disorder × time interactions are apparent on LIFE-Depression and both measures of anxiety, demonstrating that participants with anxiety disorders make greater gains in anxiety and depressive symptoms than those without. However, the absence of interaction for manic/hypomanic symptoms shows equivalent gains irrespective of the presence of a comorbid anxiety disorder.

The treatment impact on secondary outcome measures is presented in Table 2. Significant time effects show improvements on measures of global functioning, social functioning, medication compliance, dysfunctional attitudes, and coping style. The significant main effects for the presence of an anxiety disorder confirm that the anxiety disorder group fares worse in a number of areas. However, the absence of an anxiety disorder × time interaction on any secondary outcome measure shows that the participants make equivalent treatment gains irrespective of the presence of a comorbid anxiety disorder.

**Table 2:** **Treatment impact on secondary outcome variables based on anxiety disorder comorbidity**

	No anxiety disorder		Comorbid anxiety disorder		Time effect		Anxiety disorder effect		Time × anxiety disorder interaction	
	Baseline	18 months	Baseline	18 months						
	***M*** (SD)	***M*** (SD)	***M*** (SD)	***M*** (SD)	***F***	***p***	***F***	***p***	***F***	***p***
GAF	67.03 (10.76)	72.07 (14.26)	61.73 (10.50)	69.32 (13.43)	*25.54*	*<0.001*	*7.42*	*0.007*	0.53	0.47
SAS-Social	2.36 (1.16)	2.15 (0.83)	2.94 (1.41)	2.49 (1.25)	*8.05*	*0.005*	*10.95*	*0.001*	1.07	0.30
SAS-Overall	2.50 (0.88)	2.36 (0.82)	2.82 (0.81)	2.58 (0.73)	*5.96*	*0.016*	*6.71*	*0.01*	0.39	0.54
Compliance	1.46 (0.57)	1.66 (0.77)	1.36 (0.53)	1.51 (0.87)	*6.74*	*0.011*	*1.74*	*0.19*	0.11	0.74
DAS	130.17 (35.22)	113.57 (33.81)	140.27 (38.41)	124.57 (34.72)	*31.09*	*<0.001*	*4.76*	*0.03*	0.003	0.96
CIPM										
Stimulation reduction	1.92 (0.57)	2.18 (0.63)	1.95 (0.55)	2.25 (0.57)	*20.99*	*<0.001*	0.47	0.49	0.25	0.62
Seek professional help	2.17 (0.86)	2.28 (0.73)	2.15 (0.82)	2.20 (0.82)	0.39	0.53	0.36	0.55	0.06	0.81
Problem-directed coping	2.17 (0.60)	2.47 (0.64)	2.11 (0.59)	2.44 (0.52)	*29.25*	*<0.001*	0.18	0.67	0.21	0.65
Denial/blame	2.21 (0.51)	1.98 (0.50)	2.12 (0.54)	1.91 (0.46)	*15.20*	*0.001*	1.90	0.17	0.02	0.90

Significant effects are italicized. *CIPM* Coping Inventory for the Prodromes of Mania, *DAS* Dysfunctional Attitude Scale, *GAF* Global Assessment of Functioning, *SAS-* *Overall* Social Adjustment Scale-Global Overall Functioning, *SAS*-*Social* Social Adjustment Scale-Global Social Functioning.

For comparison of the treatment conditions, the analyses were repeated with psychoeducation vs. CBT treatment condition included as a covariate. None of the three-way time × anxiety disorder × treatment interaction effects approached statistical significance, suggesting that the impact of the two treatments was consistent in the presence a comorbid anxiety disorder.

Additional analyses were conducted to determine whether the effects were accounted for by differential pharmacological treatment. Full pharmacological data was available for a subsample of 114 participants. Chi-square tests show that at baseline, the participants with comorbid anxiety disorders were not more likely than those without anxiety disorders to be taking either benzodiazepines (*χ*^2^ = 1.698, *p* = 0.19) or antidepressants frequently used to treat anxiety (*χ*^2^ = 1.013, *p* = 0.31). The same held true at the 18-month follow-up assessment (benzodiazepines *χ*^2^ = 1.213, *p* = 0.27; antidepressants *χ*^2^ = 0.164, *p* = 0.69).

## Discussion

This study examined the differential impact of psychosocial interventions for BD based on whether patients had a comorbid anxiety disorder. The results showed that although those with anxiety disorders ranked more severe in several areas, the amount of improvement they gained from the treatments was equivalent or superior to the participants without anxiety disorders. They made similar gains in both CBT and psychoeducation. These results applied not only to mood symptoms and functioning but also to anxiety and a variety of secondary measures.

Among the notable findings, the participants with comorbid anxiety disorders had substantially higher anxiety levels when beginning treatment but made significant gains on anxiety that partially bridged this gap. They also made greater gains than the participants without comorbid anxiety on one measure of depression. This suggests that even when not specifically targeting anxiety, BD-specific interventions can have positive impacts on both mood and anxiety. It may be that the treatments served as a transdiagnostic exposure exercise that reduced anxiety or taught participants to better identify their symptoms. Alternatively, the treatments may have fostered improved acceptance of the disorder, reducing their anxiety about living with BD and being evaluated for it. Indeed, psychoeducation has been shown to increase acceptance of BD (Provencher et al. 2011a), while acceptance is associated with lower depression and possibly anxiety (Bussing et al. 2008). Equivalent treatment gains on dysfunctional attitudes, coping styles, and medication compliance are also encouraging since they show that comorbid anxiety does not interfere with the ability to attend to the treatment content and apply it to better manage their condition.

Given the extremely high prevalence of comorbid anxiety disorders (Merikangas et al. 2007; Hawke et al. 2013) and their negative impact on the course of illness (Hawke et al. 2013; Otto et al. 2006; Feske et al. 2000; Coryell et al. 2009), it is essential to consider comorbid anxiety in treatment research. However, there is a paucity of research examining the impact of anxiety on response to common psychological interventions for BD. This study adds to a small but growing literature on the impact of comorbid anxiety disorders on two forms of BD interventions. It also directly responds to a call for increased attention to this comorbidity set in treatment studies (Provencher et al. 2012; Provencher et al. [Bibr CR28][Bibr CR29]).

Three main approaches to the psychological treatment of the bipolar-anxiety comorbidity set have been proposed: (1) integrated, where anxiety-focused techniques are built into a BD-focused treatment; (2) sequential, where the BD and the anxiety disorder are targeted separately with disorder-specific interventions; and (3) modular, where anxiety modules are inserted into a standard BD treatment. Given that the impacts of the psychosocial interventions in the current study were equivalent irrespective of anxiety disorder status, this study provides partial support for the relevance of the sequential approach when interpreted in conjunction with promising trials of psychotherapy for comorbid anxiety (e.g., Mueser et al. 2004; Thienot et al. 2013).

Several studies have suggested that CBT-type interventions might be particularly promising for comorbid anxiety disorders (Provencher et al. [Bibr CR28][Bibr CR29]). For bipolar disorder, a randomized controlled trial of CBT for BD found that CBT was ineffective for patients with a more severe, chronic course of illness (Scott et al. 2006). Although that study did not specifically consider anxiety disorders as a severity factor, many studies have demonstrated that the course of illness is more severe in the presence of a comorbid anxiety disorder (e.g., Hawke et al. 2013; Freeman et al. 2002). Based on those findings, the efficacy of CBT might be expected to be minimal in the presence of a comorbid anxiety disorder. However, this is not the case in the current study. Although the treatment gains did not completely bridge the gap between the two groups on all measures, the magnitude of the gains was significant and substantial. Given equivalent or superior treatment gains, combined with significant improvements in anxiety symptoms, CBT and psychoeducation might be effectively combined sequentially with anxiety-focused CBT-type interventions to produce robust dual impacts. Alternatively, more specific anxiety modules might be integrated into these treatments to enhance impacts on anxiety.

Unfortunately, the combined and relative impact of each sequential treatment component cannot be evaluated within the framework of this study. Integrative research is required to examine the efficacy and optimal order of sequentially administered BD-specific and anxiety-specific interventions. Also remaining to be examined is the additional benefit gained by adding anxiety modules to existing treatments, as well as the differential impact of sequential, integrated and modular treatment approaches. Additional avenues for exploration include the mechanism by which these interventions produce reductions in anxiety symptoms and the differential impact on the various anxiety disorders.

Study limitations include the absence of a comparative treatment specifically targeting the comorbid anxiety disorder and a lack of anxiety-specific and anxiety disorder-specific measures to assess the impact on the anxiety disorder. Similarly, the sample sizes were insufficient to examine the specific impacts on the different anxiety disorders. However, the increasingly common use of transdiagnostic conceptualizations of and treatments for anxiety disorders justifies the analyses of all anxiety disorders combined (Norton and Philipp 2008). An additional limitation is that the recruitment took place primarily in academic medical centers; therefore, the results may not be fully generalizable to patients in the community.

## Conclusion

In conclusion, this study shows that individuals with comorbid anxiety disorders can make substantial gains from CBT and psychoeducation targeting BD, including reductions in anxiety symptoms. Furthermore, the gains they make in functioning, compliance, attitudes, and coping styles are equivalent to those of patients without comorbid anxiety disorders. The presence of a comorbid anxiety disorder should not be considered a deterrent to offering a patient BD-focused CBT or psychoeducation.

## Endnote

^a^The percentages do not sum to 100% due to anxiety comorbidities.

## Abbreviations

BD: Bipolar disorder
CARSM: Clinician-administered rating scale for mania
CBT: Cognitive-behavioral therapy
CIPM: Coping inventory for the prodromes of mania
DAS: Dysfunctional attitude scale
GAF: Global assessment of functioning
HAMD: Hamilton rating scale for depression
LIFE: Longitudinal interval follow-up evaluation
SAS: Social adjustment scale.
